# Young Patients’ Satisfaction Following the Correction of Adolescent Idiopathic Scoliosis in Saudi Arabia: A Cross‐Sectional Study

**DOI:** 10.7759/cureus.30058

**Published:** 2022-10-08

**Authors:** Zayed S Alzayed, Ozair B Majid, Saeed A Alqahtani, Iram Saba, Mohammed A Al Rushud, Abdullah T Eissa

**Affiliations:** 1 Orthopaedic Surgery, King Faisal Specialist Hospital and Research Centre, Riyadh, SAU; 2 Orthopaedic Surgery, Sultan Bin Abdulaziz Humanitarian City, Riyadh, SAU; 3 Orthopaedic Surgery, King Faisal Medical City, Abha, SAU; 4 Research, Sultan Bin Abdulaziz Humanitarian City, Riyadh, SAU

**Keywords:** posterior spinal instrumented fusion, patient satisfaction, quality of life, scoliosis, srs-22, adolescent idiopathic scoliosis (ais)

## Abstract

Introduction: Posterior spinal instrumented fusion remains the mainstay treatment for adolescent idiopathic scoliosis (AIS) with acceptable post-operative patient satisfaction. However, in Saudi Arabia, patient satisfaction after surgical management for AIS has not been thoroughly studied. The purpose of this study was to determine patient satisfaction and quality of life using the Scoliosis Research Society-22r (SRS-22r, the most recent version) questionnaire after surgical correction of AIS in Saudi Arabia.

Methods: A retrospective study was conducted that included patients who underwent posterior spinal instrumented fusion for AIS from January 1995 through December 2015. We included 115 patients (both males and females) in our study. We used the Arabic version of the SRS-22r questionnaire that was completed through telephonic interviews. Data collected were then analyzed using SPSS Statistics, version 23.

Results: The mean age of our patients at the time of surgery was 15.0 ± 2.6 years and the average time from surgery to interview was 9.4 ± 4.7 years. A positive response was recorded in all domains including pain, function, mental health, and self-image. Furthermore, 76.5% of the patients were satisfied with their management outcome and 81.7% of the patients reported no complications.

Conclusion: Surgical correction of AIS improved the quality of life of our patients that was assessed using the Arabic version of the SRS-22r questionnaire. Apart from overall patient satisfaction, positive responses were recorded in all four domains of the SRS-22r questionnaire.

## Introduction

Adolescent idiopathic scoliosis (AIS) is one of the most common spinal deformities with a prevalence of 1%-3% [1]. Although there are no recent studies, to our knowledge, regarding AIS epidemiology in Saudi Arabia, reported epidemiological patterns of scoliosis are comparable to international findings [2]. To stop the progression of the curve, surgical intervention in terms of posterior spinal fusion (PSF) might be indicated in patients with curves greater than 45° [3].

Self-reported postoperative outcomes are influenced by several factors that include but are not limited to, age, ethnicity, self-image, sociocultural issues, and sex [4]. Also, patient satisfaction with surgical outcomes is one of the most important factors [5]. The importance of patient satisfaction is reflected in the reduced risk of malpractice allegations and increased productivity of healthcare professionals and the overall productivity of hospitals. Unfortunately, little is known about patient satisfaction following orthopedic surgeries in Saudi Arabia [6].

The Scoliosis Research Society (SRS) proposed a questionnaire to assess patients’ perceptions and satisfaction with scoliosis treatment [7]. The SRS-22 questionnaire is widely used to evaluate surgical outcomes in AIS. Haidar et al. translated the questionnaire into Arabic. The Arabic version of the SRS-22r (SRS-22r being the revised and most recent version) questionnaire was found to be reliable and valid [8]. As far as we know, patient satisfaction after surgical management of AIS in Saudi Arabia has not been studied. To the best of our knowledge, the present study will be the first to address questions related to the satisfaction of AIS patients undergoing PSF. In addition, we focused on studying the characteristics that affect AIS patient satisfaction after surgery.

## Materials and methods

Subjects and sample size

This retrospective cross-sectional study was carried out at our hospital King Faisal Specialist Hospital and Research Centre, Riyadh. We investigated the satisfaction of male and female Saudi patients 10-20 years of age, who underwent posterior spinal fusion and instrumentation from January 1995 through December 2015. Simple random sampling was used to select the patients for the study. Using a 95% confidence interval of the width of no more than 10% and assuming a targeted proportion of 50%, the minimum sample size required was 96 participants. The sampling process was performed using an electronically generated list of medical record numbers of all eligible patients who met the inclusion and exclusion criteria. The participants were randomly selected from the list using electronic data spreadsheet software (Excel 2016 for Mac; Microsoft, Redmond, WA).

Males and females aged 10-20 years were included in the study. Patients diagnosed with idiopathic adolescent scoliosis and who underwent surgery only in our centre were included. Patients with other forms of early onset scoliosis were excluded. Those with other medical conditions or syndromes affecting general health were also excluded. Furthermore, patients with mental or physical disabilities that barred their ability to complete a telephone interview were excluded from the study.

Participants provided verbal informed consent at the beginning of each telephone interview. Participants were informed of their right to withdraw from the study at any stage or to have their data excluded from the analysis, and they could interrupt the interview at any time. The interviews were not recorded, and participants did not receive compensation of any kind.

Questionnaire

Owing to the scarcity of studies in this area in Saudi Arabia, we used the Arabic version of the Scoliosis Research Society-22r questionnaire that composed of 22 items divided into five domains: pain, function, self-image, mental health and satisfaction, and a total score [7]. Each domain score ranged from 1 to 5, with higher scores indicating better outcomes. The Arabic version of the SRS-22r questionnaire was found to be reliable and reproducible in Arabic-speaking patients with idiopathic scoliosis [8]. The first section, which is not included in the original SRS-22r, principally asks for demographic data.

After the eligibility of a patient was confirmed, the questionnaire was completed by interviewing the participants by telephone, and responses were recorded in a customized REDCap (Research Electronic Data Capture) database. Four interviewers were involved. At the beginning of the phone call, a standard statement was read to the prospective subject to obtain one's consent to being asked the questionnaire; participation was completely voluntary. This consent was taken orally and documented in the REDCap record for the patient by clicking on an associated box and entering the date and time. All interviewers used the exact wording when asking the questions. To prevent non-response bias, a set of measures were implemented, including proper selection and training of the interviewers, and three attempts were made at different parts of the day in the case of failure to reach a patient by telephone. Finally, the study was completed with 115 patients.

All data acquired from the questionnaire that was answered by participants was stored in a password-protected PC in a locked room. Moreover, the confidentiality of the study participants’ data and their anonymity were ensured at all times. We did not encounter any issues regarding keeping the patient’s data confidential.

Data analysis

IBM SPSS Statistics, version 23 (IBM Corp., Armonk, NY) was used for all statistical analyses. Continuous variables like age and follow-up years were presented as means ± standard deviations while categorical variables were presented as frequencies (%). The prevalence of the subjects who opted for the five options in the SRS-22r questionnaire was presented as frequencies (%). The SRS-22r questionnaire was divided into five sections based on questions related to “pain”, “function”, “self-image’, “mental health” and the “satisfaction/dissatisfaction with management”, and the responses were scored as “1 to 5” with “5” indicating the best outcome. The subjects were then categorized based on “gender”, “age at surgery” and “follow-up years” and the average score for each of the five sections in the SRS-22r questionnaire was presented as the mean ± standard deviation. The difference in the average scores in the groups mentioned was calculated using an independent Student’s t-test, and in all statistical tests, p<0.05 was considered significant. All the figures presented in this article were plotted using Microsoft Excel.

## Results

Demographic characteristics of the study participants

We analyzed 115 patients, of whom 103 were females and 12 were males. The mean age of our population at the time of surgery was 15.0 ± 2.6 years, while the mean age at the time of interviews was 24.5 ± 5.1 years with an average surgery to interview interval of 9.4 ± 4.7 years. Most of our patients lived in an urban setting (65.22%, 75 patients) while only 26.08% lived in rural homes (30 patients), and the remaining 8.69% (10 patients) chose not to answer this question. A total of 65.22% (75) patients completed their university education after the surgery and 26.08% (30 patients) completed their high school diplomas; one subject was illiterate (0.87%) and nine patients did not answer this question (7.82%). In terms of marital status, 63.47% (73) of patients were not married at the time of the interview; 27.82% (32) were married, 1.73% (2) were divorced and 6.95% (8) opted out of the question. Most subjects were students with 46.95% (54) of the population being actively enrolled in undergraduate or post-graduate studies. A total of 25.21% (29) of subjects were unemployed and 20% (23) were employed, while 7.83% (9) did not answer the question (Table 1).

**Table 1:** Demographic characteristics of study participants ^a^Continuous variables are presented as means ± standard deviations. Categorical variables are presented as frequencies (percentages).

Variable	All patients (n=115)
Age at the time of surgery (years)^a^	15.0 ± 2.6
Age at the interview (years)^a^	24.5 ± 5.1
Follow-up (years)^a^	9.4 ± 4.7
Sex	
Female	103 (89.6)
Male	12 (10.4)
Geographical zones	
Middle	59 (51.3)
Western	17 (14.7)
Eastern	13 (11.3)
North	11 (9.5)
South	7 (6.08)
Rural/urban	
Urban	75 (65.2)
Rural	30 (26.08)
Marital status	
Not married	73 (63.4)
Married	32 (27.8)
Divorced	2 (1.7)
Education	
University	75 (65.2)
High school	30 (26.08)
Illiterate	1 (0.86)
Employment status	
Employed	23 (20)
Not employed	29 (25.2)
Student	54 (46.9)

Patient responses to the SRS-22r questionnaire items

The responses to the five sections of the SRS-22s questionnaires are given in Table 2. The crude responses were then grouped as positive and negative responses, analyzed, and interpreted the results.

**Table 2:** Patient responses to the SRS-22r questionnaire items SRS-22r, Scoliosis Research Society-22r The data are presented as the frequency (percentage) of the patients opting for different responses to the SRS-22r questionnaire items.

Domains and responses				
Pain				
Q1. Which one of the following best describes the amount of pain you have experienced during the past 6 months?				
Severe	Moderate to severe	Moderate	Mild	None
8 (7)	13 (11.3)	25 (21.7)	28 (24.3)	41 (35.7)
Q2. Which one of the following best describes the amount of pain you have experienced over the last month?				
Severe	Moderate to severe	Moderate	Mild	None
6 (5.2)	7 (6.1)	20 (17.4)	30 (26.1)	52 (45.2)
Q8. Do you experience back pain when at rest?				
Very often	Often	Sometimes	Rarely	Never
6 (5.2)	16 (13.9)	43 (37.4)	21 (18.3)	29 (25.2)
Q11. Which one of the following best describes your pain medication use for back pain?				
Narcotics daily	Narcotics weekly or less	Non-narcotics daily	Non-narcotics weekly or less	None
1 (0.9)	0 (0)	0 (0)	3 (2.6)	93 (80.9)
Q17. In the last 3 months, have you taken any days off of work or school because of back pain?				
4 or more days	3 days	2 days	1 day	0 days
7 (6.1)	2 (1.7)	3 (2.6)	2 (1.7)	101 (87.8)
Function				
Q5. What is your current level of activity?				
Bedridden	No activity	Light	Moderate	Full activity
0 (0)	3 (2.6)	23 (20)	18 (15.7)	71 (61.7)
Q9. What is your current level of work/school activity?				
0% normal	25% normal	50% normal	75% normal	100% normal
3 (2.6)	2 (1.7)	15 (13)	33 (28.7)	62 (53.9)
Q12. Does your back limit your ability to do things around the house?				
Very often	Often	Sometimes	Rarely	Never
7 (6.1)	8 (7)	37 (32.2)	17 (14.8)	46 (40)
Q15. Are you and/or your family experiencing financial difficulties because of your back?				
Severely	Moderately	Mildly	Slightly	None
2 (1.7)	5 (4.3)	9 (7.8)	4 (3.5)	95 (82.6)
Q18. Does your back condition limit your going out with friends/family?				
Very often	Often	Sometimes	Rarely	Never
6 (5.2)	22 (19.1)	71 (61.7)	11 (9.6)	5 (4.3)
Self-Image				
Q4. If you had to spend the rest of your life with your back shape as it is right now, how would you feel about it?				
Very unhappy	Sometimes unhappy	Neither	Sometimes happy	Very happy
8 (7)	12 (10.4)	19 (16.5)	31 (27)	45 (39.1)
Q6. How do you look in clothes?				
Very bad	Bad	Fair	Good	Very good
3 (2.6)	5 (4.3)	18 (15.7)	26 (22.6)	63 (54.8)
Q10. Which of the following best describes the appearance of your trunk				
Very poor	Poor	Fair	Good	Very good
1 (0.9)	10 (8.7)	22 (19.1)	26 (22.6)	56 (48.7)
Q14. Do you feel that your back condition affects your personal relationships?				
Severely	Moderately	Mildly	Slightly	None
6 (5.2)	4 (3.5)	5 (4.3)	15 (13)	84 (73)
Q19. Does your back condition limit your going out with friends/family?				
Very often	Often	Sometimes	Rarely	Never
7 (6.1)	13 (11.3)	37 (32.2)	30 (26.1)	28 (24.3)
Mental Health				
Q3. During the past 6 months, have you been a very nervous person?				
All the time	Most of the time	Sometimes	Very less	None
5 (4.3)	17 (14.8)	44 (38.3)	23 (20)	26 (22.6)
Q7. In the past 6 months, have you felt so down in the dumps that nothing could cheer you up?				
Very often	Often	Sometimes	Rarely	Never
9 (7.8)	6 (5.2)	31 (27)	21 (18.3)	48 (41.7)
Q13. Have you felt calm and peaceful during the past 6 months?				
Never	Rarely	Sometimes	Often	All the time
7 (6.1)	11 (9.6)	34 (29.6)	46 (40)	17 (14.8)
Q16. In the past 6 months, have you felt downhearted and blue?				
Very often	Often	Sometimes	Rarely	Never
1 (0.9)	8 (7)	39 (33.9)	17 (14.8)	49 (42.6)
Q20. Have you been a happy person during the past 6 months?				
Never	Rarely	Sometimes	Often	All the time
2 (1.7)	8 (7)	29 (25.2)	51 (44.3)	25 (21.7)
Satisfaction/dissatisfaction with management				
Q21. Are you satisfied with the results of your back management?				
Very unsatisfied	Unsatisfied	Neither	Satisfied	Very satisfied
5 (4.3)	7 (6.1)	15 (13)	33 (28.7)	55 (47.8)
Q22. Would you have the same management again if you had the same condition?				
Definitely not	Probably not	Not sure	Probably yes	Definitely yes
9 (7.8)	4 (3.5)	9 (7.8)	19 (16.5)	73 (63.5)

**Figure 1:**
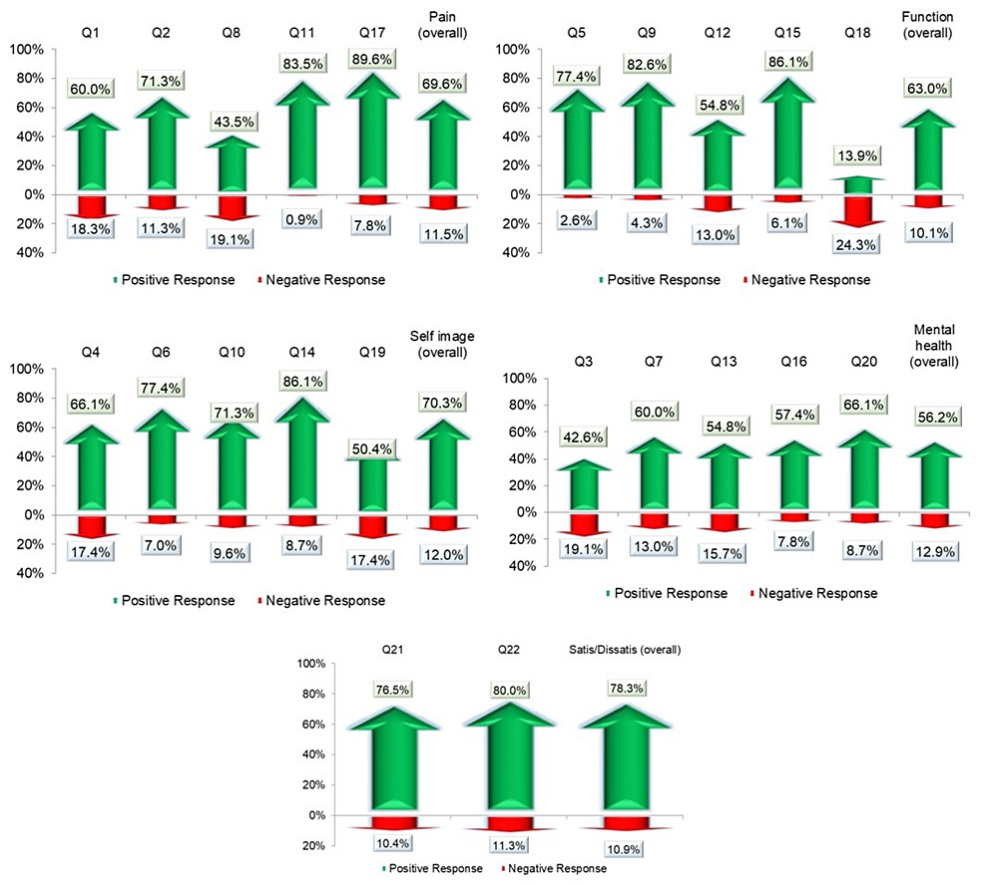
Positive and negative responses to SRS-22r questionnaire items provided by the patients SRS-22r, Scoliosis Research Society-22r Here the responses ‘1’ and ‘2’ are together shown as ‘negative responses’ and responses ‘4’ and ‘5’ are shown as ‘positive responses’. These 22 questions were further categorized into five sections ‘pain’, ‘function’, ‘self-image’, ‘mental health’, and ‘satisfaction/dissatisfaction with management.

The overall response to the questions related to pain was positive in 69.6% of the patients, and 11.5% gave negative responses to the same questions. The remaining patients did not answer the question. Similarly, the overall positive response to functional assessment was 63%, a positive response to self-image was documented in 70.3% and an overall positive response to mental health-related questions was seen in 56.2%. Noticeably, overall satisfaction from the surgery was documented in 76.5% of the patients while at the same time, 80% of subjects agreed that, given the option, they would still choose the same surgical management again if they had the same condition (Figure 1).

SRS-22r scores according to sex, age at surgery and follow-up years

**Figure 2:**
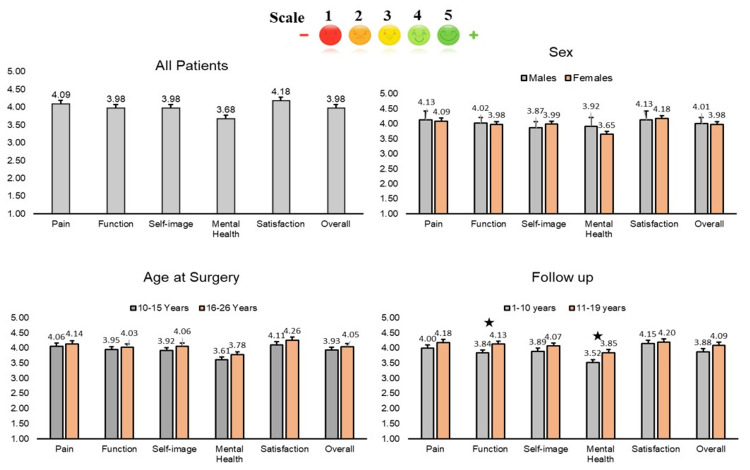
Average scores on a scale of 1-5 calculated for the five sub-categories of the SRS-22r questionnaire SRS-22r, Scoliosis Research Society-22r The star symbol signifies p-values <0.05.

Patient responses to the SRS-22r questionnaire were scored as “1 to 5” with “5” indicating the best outcome, and then the average scores for each section of the questionnaire were tabulated (Table 3). In all domains of pain, function, self-image, mental health and satisfaction, the mean score on a scale of 1-5 was more than 3, and the overall mean score was 3.98 ± 0.6, denoting excellent patient satisfaction with the outcome of surgery. The lowest mean score was 3.68 ± 0.8 documented in the domain of mental health status. The data were divided by sex, age at surgery, and follow-up years; the average scores are plotted as bar graphs in Figure 2.

**Table 3:** Mean patient scores of the responses to the SRS-22r questionnaire SRS-22r, Scoliosis Research Society-22r Data are presented as means ± standard deviations. p-values were calculated by an independent t-test, and p<0.05 was considered significant.

SRS-22r domains		Sex			Age at surgery			Follow-up years		
	All patients (115)	Males (12)	Females (103)	p-value	Age at surgery 10-15 years (67)	Age at surgery 16-26 years (48)	p-value	Follow up 1-10 years (58)	Follow up 11-19 years (57)	p-value
Pain	4.09 ± 0.8	4.13 ± 0.9	4.09 ± 0.7	0.84	4.06 ± 0.8	4.14 ± 0.7	0.6	4.00 ± 0.7	4.18 ± 0.8	0.21
Function	3.98 ± 0.6	4.02 ± 0.6	3.98 ± 0.6	0.84	3.95 ± 0.7	4.03 ± 0.5	0.49	3.84 ± 0.6	4.13 ± 0.6	0.02
Self-image	3.98 ± 0.9	3.87 ± 0.7	3.99 ± 0.9	0.66	3.92 ± 0.9	4.06 ± 0.8	0.41	3.89 ± 0.9	4.07 ± 0.8	0.28
Mental health	3.68 ± 0.8	3.92 ± 0.9	3.65 ± 0.8	0.29	3.61 ± 0.7	3.78 ± 0.9	0.27	3.52 ± 0.7	3.85 ± 0.8	0.03
Satisfaction/dissatisfaction	4.18 ± 1.0	4.13 ± 1.0	4.18 ± 1.0	0.85	4.11 ± 1.0	4.26 ± 0.9	0.43	4.15 ± 0.9	4.2 ± 1.0	0.77
Overall	3.98 ± 0.6	4.01 ± 0.5	3.98 ± 0.6	0.85	3.93 ± 0.5	4.05 ± 0.6	0.25	3.88 ± 0.5	4.09 ± 0.6	0.05

There was no statistically significant difference between the mean scores of each domain and gender or age at the time of surgery; however, there was a significant difference between the functional score and follow-up for more than 10 years in comparison to less than 10 years, with a p-value of 0.02. There was also a statistically significant difference in the mental health of patients who had surgery more than 10 years ago in comparison to those who had surgery within the last 10 years, with a p-value of 0.03.

Complications

**Figure 3:**
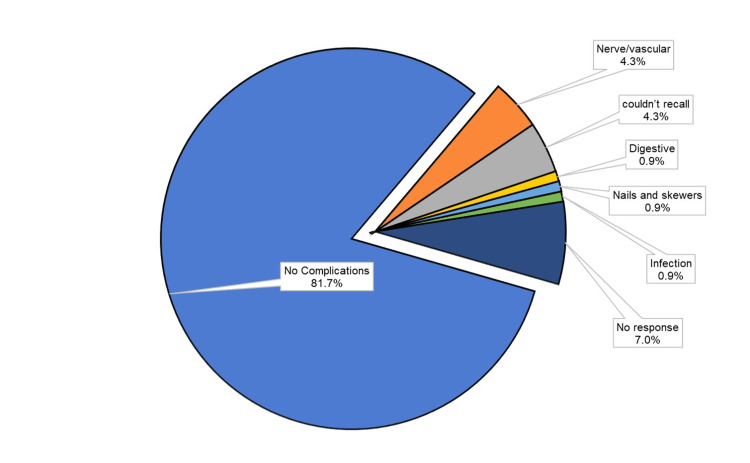
Complications reported by patients after surgery

Most patients (81.7%) reported no complications at all while other complications such as infections or neurovascular conditions were transient and patients recovered without any long-term sequelae. The complications reported are plotted as a pie chart in Figure 3.

## Discussion

We studied patient satisfaction after the surgical management of AIS in Saudi Arabia followed up over 9.4 ± 4.7 years, using the SRS-22r questionnaire. We studied responses on multiple domains such as pain, function, self-image, mental health and overall satisfaction that were self-reported by the patients. Although there have been multiple studies about short- and long-term patient satisfaction after different methods of surgical management of AIS, our study is the first of its kind in Saudi Arabia.

In our study, the mean score in all domains, on a scale of 1-5, was more than 3. Furthermore, in our survey, 76.5% of the patients were satisfied with the management of AIS indicating the good outcome of surgical treatment of AIS.

Akazawa et al. studied 256 patients surgically treated for AIS using the SRS-22 questionnaire and Roland-Morris Disability Questionnaire. In their study, the mean follow-up period was 31.5 years. The pain and mental health scores were 4.3 and 3.9, respectively, whereas, function and self-image scores were reported to be 4.3 and 3.0, respectively. They concluded that surgery for AIS has good long-term outcomes [9]. In a similar survey of 254 patients by Chau et al., the quality of life was studied over 30 years after surgery using the SRS-22 questionnaire. They noticed that in the first five years of follow-up, all domain scores remained relatively stable and later on from 9 years to more than 30 years post-surgery, all the domain scores started to gradually decline [10]. They further documented that self-image scores were among the lowest throughout the years after surgery.

Aghdasi et al. in a quantitative meta-analysis of seven studies, using the SRS-22 questionnaire, after surgical correction of AIS at 24- and >60-month follow-ups noted significant improvement in all domain scores. At the >60-month follow-up, all domain scores fetched high scores except function. They further concluded that spinal fusion for AIS improved quality of life in medium- and long-term follow-ups [11]. Marks et al. in their study of 547 patients found no significant difference in postoperative domain scores between males and females [12]. In our study, we also found no difference in the mean score of each domain concerning gender. Similarly, Fernandes et al. studied 38 AIS patients using the SRS-22 questionnaire. Significant changes in the domains of self-image and mental health were encountered between the preoperative and postoperative periods, whereas pain and activity domains remained largely unchanged. They further emphasized a thorough evaluation of the patients before a surgical intervention [13].

In our study, 7% of patients had major or minor complications resolving spontaneously without any major intervention or sequelae. Bastrom et al. studies 1486 patients with AIS utilizing both preoperative and postoperative SRS-22 questionnaires. Both major and minor complications were reported in 15% of patients at the two-year follow-up. They also studied the effect of complications on the domains and noticed an impact on domain scores due to both major and minor complications [14]. Marks et al. in their study of 1757 patients documented the rate of surgical site infection to be 1.6% after surgery for AIS. The majority of these patients recovered and became pain-free during the follow-up [15].

The impact of posterior spinal instrumented fusion on quality of life has been extensively studied. Moreover, the correlation between domain scores and variables such as sex and follow-up years after surgery is also reported in the literature [16-19]. In our study, we also looked at the correlation between mean scores of each domain and age at the time of surgery. We divided the patients into two groups. One group had surgery at 10-15 years of age and the other group had surgery at 16-20 years of age. There was no statistically significant correlation between age at the time of surgery and the mean score of each domain.

Our study also has some limitations that should be mentioned here. This was a retrospective study where we had less control over procedures and there was a possibility of missing data. Also, given the nature of our study and research tools, we expected a recall bias. However, to minimize this effect, we extensively reviewed patient records in search of unreported complications such as infections or neurovascular injuries. We assume that the surgeon’s experience is an important factor affecting the outcome. We did not take into account this factor in our study. Our average follow-up of patients was 9.4 years. It is necessary to have a long-term follow-up to assess the quality of life after the surgery over a longer period.

## Conclusions

In our study, which to our knowledge is the first of its kind in Saudi Arabia, we have reported on the mid-term outcome of surgical treatment of AIS using the SRS-22r questionnaire. We conclude that a good outcome was achieved with surgery when taking all domains such as pain, function, self-image and mental status into account. The majority of patients were satisfied with the outcome of the surgery. Further studies focusing on the long-term outcome of surgery for AIS should be done to shed more light on the outcome of such surgeries. Moreover, surgeons must thoroughly evaluate the benefits and possible complications before the procedure.
